# Supplementary material for: Predicting the ritonavir crisis by revisiting the polymorph landscape with crystal structure prediction and form 4 structure solution
Source: Commun Chem. 2025 Dec 22;8:404. doi: 10.1038/s42004-025-01814-6 (PMC12722319; doi:10.1038/s42004-025-01814-6)
Supplement: Supplementary file 2 — Supporting Information document [file 42004_2025_1814_MOESM2_ESM.pdf]

Supporting Information. Predicting the ritonavir crisis by revisiting the polymorph landscape with crystal structure prediction and form 4 structure solution

Luca Iuzzolino,[a] Andrew W. Kelly,[b] Mohammad T. Chaudhry,[b] Cristian Jandl,[c] Danny Stam,[c] Alfred Y. Lee[b]

a. Modeling & Informatics, Discovery Chemistry, Merck & Co., Inc., Rahway NJ 07065, USA
b. Analytical Research & Development, Merck & Co., Inc., Rahway NJ 07065, USA
c. ELDICO Scientific AG, Switzerland Innovation Park Basel Area, Hegenheimermattweg 167A, 4123 Allschwil, Switzerland

Supplementary Note 1. Data choice for form 3.

We decided to consider YIGPIO06 (*P*1, Z’ = 4)[1] as the best representative of form 3. One significant factor is that other reported datasets (*e.g.*, *C*2 structure YIGPIO04[2], where Z’ = 1) likely have incorrect space group choices and unrealistic data modelling (discussed in detail in the paragraphs below). Additionally, YIGPIO04 and YIGPIO06 are the same structure, just modelled differently, as seen through their respective reduced cells, which are nearly identical.[3] As there are conflicting results in the literature and an incorrect choice for the space group of ritonavir form 3 (*C*2 vs. *P*1) would affect the accuracy and detailed solid-form landscape, it was crucial to address them.

The discrepancy in the published structure of ritonavir form 3 may be attributed to the challenge of determining an appropriate space group due to the rotational freedom within ritonavir, resulting in disordered structures. Initially, when studying YIGPIO06, the authors[1] used an in-house diffractometer and determined a *C*2 monoclinic crystal system with cell dimensions that were nearly identical to those of YIGPIO04, which was also modeled in *C*2. However, when YIGPIO06 was subsequently collected using synchrotron radiation, which provided significantly better resolved data compared to the in-house diffractometer, the structure was found to be in the *P*1 space group. The utilization of synchrotron data likely facilitated the deconvolution of overlapping diffraction peaks, resulting in stronger and more accurate data, which was able to give the realistic Z’ = 4 solution.

Additionally, it is not surprising that there was difficulty in selecting the appropriate space group, considering the similarity between the disordered *C*2 (Z’ = 1) structure and the *P*1 (Z’ = 4) structure (see Supplementary Figure 1). When observed along the a-axis, the disorder in YIGPIO04 (shown in pink) aligns well with two chemically independent molecules of ritonavir in YIGPIO06 (highlighted in purple for clarity). The starkest differences are in the thermal parameters for YIGPIO06, which are more appropriate and significantly less oblong and directional than in YIGPIO04. In fact, some of the thermal ellipsoids in YIGPIO04 have such high aspect ratios that

numerous A- and B- level alerts were seen in the corresponding CheckCIF reports, implying a poor refinement model.[3,4] In contrast, YIGPIO06 has thermal parameters that appropriate (more uniform in shape) and are realistic (no extremely long or short ellipsoids with large directional preference). Furthermore, the *P*1 structure YIGPIO04 has a lower R_1 (8.78%) than the *C*2 structure YIGPIO04 (12.07%), further implying the former is the more likely correct solution.

The presence of considerable disorder in form 3, which was grown through melt crystallization, was unexpected. It has been postulated that crystal growth from the melt has a high propensity for forming large Z' structures,[5] which can be challenging to obtain through traditional solvent-based crystallization methods. In fact, even in the *P*1 (Z' = 4) structure, there is still disorder in several groups that needed to be modelled. Thus, based on the aforementioned evidence (space group choices and disorder modelling), the structure YIGPIO06 is the most realistic dataset for ritonavir form 3.

Unfortunately, as the most realistic structure for ritonavir Form 3 is the Z' = 4 structure, CSP for this structure is unfeasible. Although the CSP search space included *C*2 and *P*1 structures, the higher symmetry *C*2 and *P*1 versions of form 3 would be too high in energy to be captured by CSP.

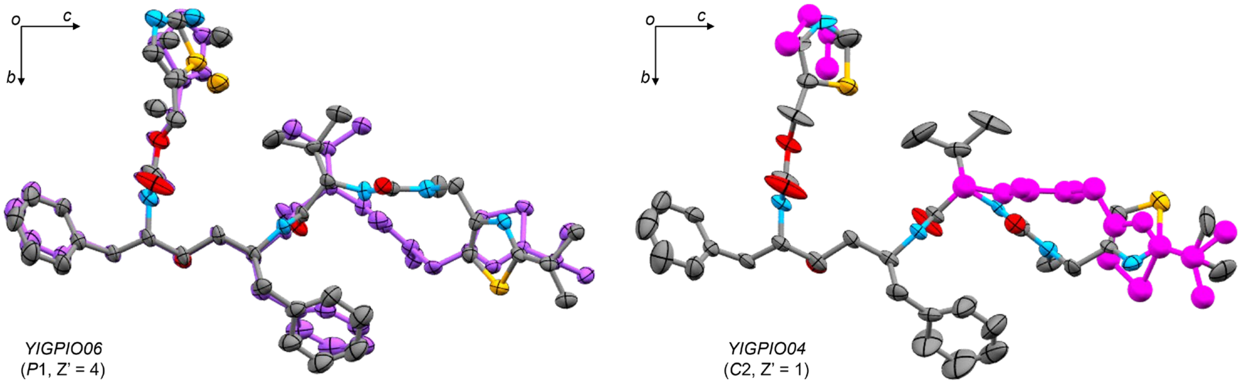

Supplementary Figure 1: View of structural motifs in YIGPIO06 and YIGPIO04 looking down the a-axis. Color code: Grey (carbon), red (oxygen), blue (nitrogen) orange (sulfur), purple (crystallographically inequivalent molecule for ritonavir found in YIGPIO06), and pink (disordered motif found in YIGPIO04). Thermal parameters given at the 50% confidence level. Hydrogen atoms omitted for clarity.

Supplementary Note 2. CSP on ritonavir.

The crystal structure prediction (CSP) study on ritonavir was performed using GRACE 2.8.[6] First, a tailor-made force field (TMFF)[7] was fitted to *ab initio* data generated by dispersion-corrected density functional theory (DFT-D).[8] This TMFF uses fixed point charges.[7] The PBE functional[9] and the two-body Neumann-Perrin (NP)[10] empirically-fitted dispersion correction were employed. The NP correction has been developed at Avantgarde Materials Simulation, while VASP[11-13] or TURBOMOLE[14] were used for DFT. The TMFF is needed to obtain a preliminary ranking of the relative E_{latt} values for the large number of crystal structures commonly generated by a CSP search, which could not be all feasibly calculated with quantum mechanics.[15] The accuracy of the TMFF was then further enhanced by performing a preliminary CSP on ritonavir; this process is known as 'back-fitting.'[16,17] The CSP process in GRACE consists of three steps for each set of Z' values and set of space groups that were considered. In this study, only a Z' value of 1 was covered; as ritonavir is a chiral molecule crystallized enantiopure, only the Sohncke space groups were considered.[18] Z' = 1 crystal structures with generated in 21 space groups (P1, $P2_1$, C2, $P2_12_12$, $P2_12_12_1$, $C222_1$, $P4_1$, I4, $I4_1$, $P4_12_12$, $P3_1$, R3, $P3_12_1$, $P6_1$, $P6_122$, $P4_3$, $P3_2$, $P6_5$, $P4_32_12$, $P6_522$, $P3_221$) which cover ~99.8% of CSD[19] homochiral crystal structures with one molecule in the asymmetric unit. As a first CSP step, crystal structures were generated by a Monte Carlo parallel tempering algorithm using the TMFF. Subsequently, the most promising candidates after the search were optimized with DFT-D with loose convergence criteria, and finally, the most stable crystal structures underwent a final optimization with tighter convergence settings. The first and second steps are statistically controlled to guarantee their completeness, and they were considered converged when 99% of the structures within some target energy windows were identified. How the energy windows are identified, and the completeness level estimated, are described elsewhere.[16] All the DFT-D calculations were carried out at the PBE-NP level of theory. The DFT calculations, performed with VASP, used a plane wave cutoff energy of 520 eV and a k-point spacing of approximately 0.07 $Å^{-1}$. In the intermediate CSP step, lattice energy optimizations were converged to within at least 0.02 Å for atomic displacements, 0.001 $kcal{\cdot}mol^{-1}$ per atom for energy changes, 7.0 $kcal{\cdot}mol^{-1}{\cdot}Å^{-1}$ for the atomic forces, and 15.0 kbar for cell stress. In the final CSP step, tighter convergence settings were used, and the optimizations were converged to within at least 0.003 Å for atomic displacements, 0.00025 $kcal{\cdot}mol^{-1}$ per atom for energy changes, 0.7 $kcal{\cdot}mol^{-1}{\cdot}Å^{-1}$ for the atomic forces and 1.0 kbar for cell stress. Since form 3 could not be generated by CSP, as it is a Z' = 4 crystal structure,[1] it was optimized from the single crystal X-ray diffraction solution with the same DFT-D settings as the final CSP step to place it on

the crystal energy landscape. More detail can be found in the section above and in the main manuscript.

Although the PBE-NP lattice energies have proven successful in several CSP studies, a more accurate energy ranking method called TRHu(ST)23 has recently been reported and validated.[20] For all the crystal structures coming out of the CSP process (including optimized form 3) the TRH(ST)23 lattice energies were calculated using GRACE 3.1 and its interface with the all-electron code FHI-Aims.[21] The level of theory and corrections included in the TRHu(ST)23 lattice energy method are described elsewhere, but in summary they include recalculating the crystalline energies at the PBE0-MBD-NL[22-24] level of theory and adding a post-Hartree Fock monomer correction computed with the MP2D method.[25]

Supplementary Note 3. Structure solution of form 4

3.1 Preparation of ritonavir form 4

The replication of form 4 of ritonavir posed unanticipated challenges. Despite following the experimental procedures outlined in the patent literature,[26] utilizing an EasyMax reactor to regulate temperature and heating/cooling rates, and a four dram vial, only form 1 was produced. Since high supersaturation is required to nucleate form 4, the capillary crystallization technique[27] was exploited using a 1.0 mm glass capillary from Hampton Glass 0500, borosilicate glass which[27] yielded a mixture of form 1 and form 4, as confirmed by powder X-ray diffraction (XRPD) and differential scanning calorimetry (DSC) analyses, aligning with the reported outcomes in the patent and the literature.[28] This sample was provided to three-dimensional electron diffraction (3D ED) experts to perform structure solution.

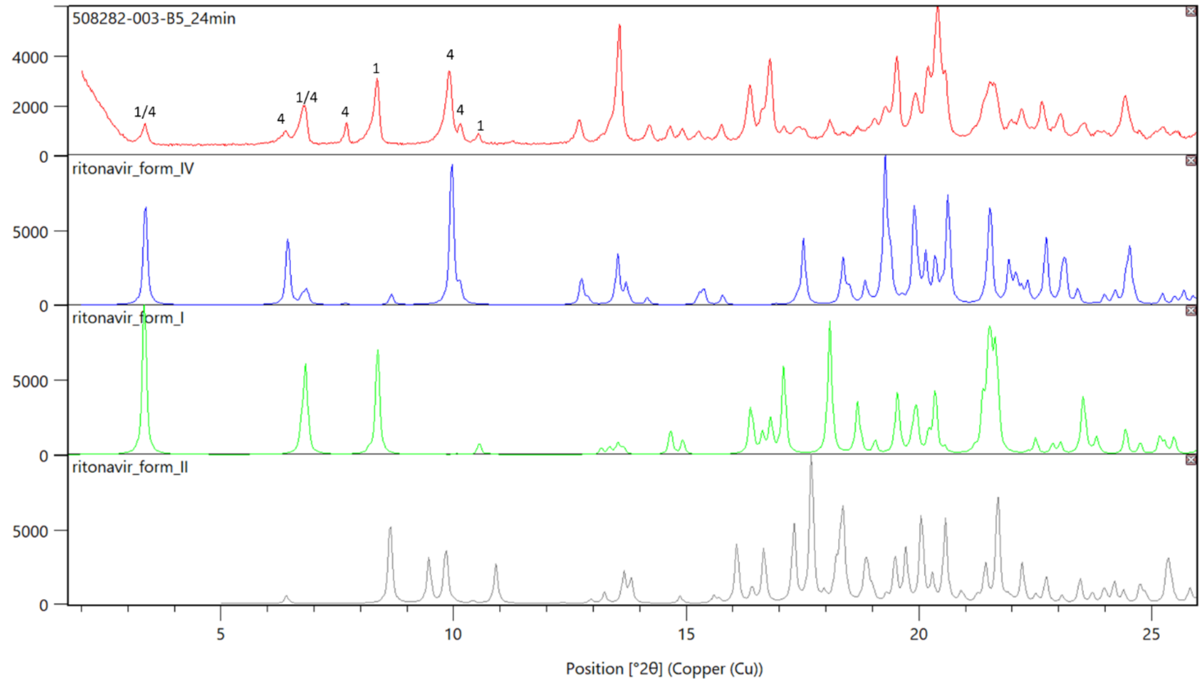

Supplementary Figure 2: The powder X-ray diffraction (XRPD) results for a mixture of ritonavir form 4 and form 1 are presented as the red trace. The XRPD data for ritonavir form 4 was sourced from Morissette e*t al.*[28] Reference patterns for ritonavir form 1 (shown as the green trace) and form 2 (shown as the grey trace) were created using the Mercury software, based on their respective crystal structures YIGPIO02 and YIGPIO03. Each peak corresponding to the different forms in the mixed sample is clearly labeled.

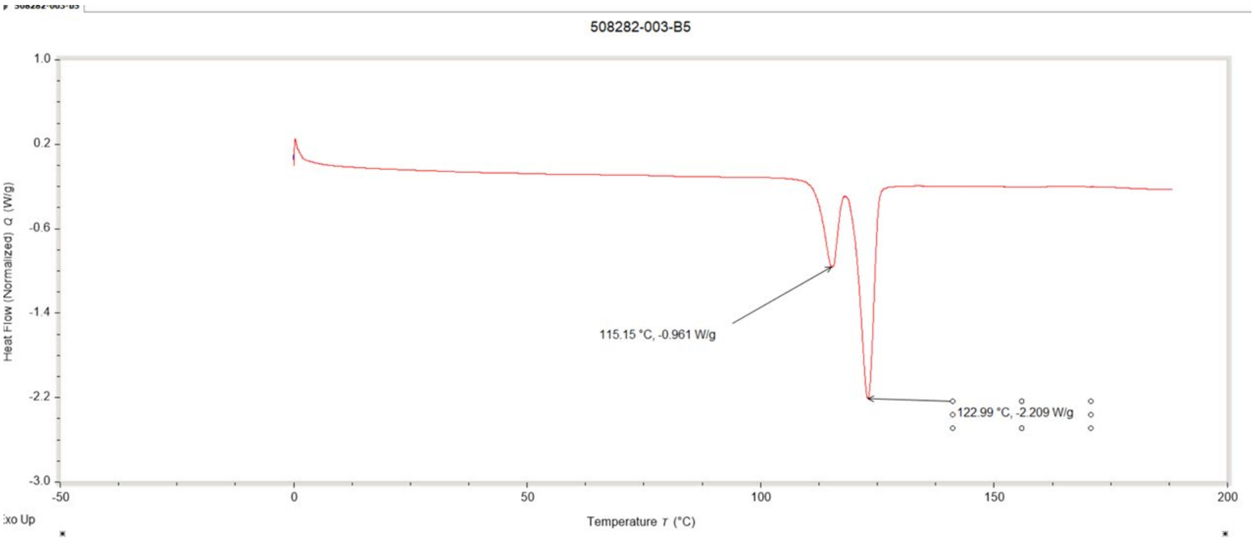

Supplementary Figure 3: Differential Scanning Calorimetry (DSC) of the mixture of forms 1 and 4. Two melting points are observed. The peak at 115 °C was assigned to the melt of form 4. The peak at 122 °C was assigned to form 1.[28]

3.2 Looking for form 4 in the CSP data

The powder X-ray pattern of form 4 from a ritonavir solid form patent[26] was digitized using WebPlotDigitizer.[29] Then, the crystal structures generated by CSP were evaluated against the 'digitized' XRPD pattern of form 4 using a cross-correlation function method available in GRACE.[30] In each comparison, a global temperature factor, March-Dollase preferred orientation parameters[31] and the cell parameters of the CSP-generated structure were adjusted to maximize the similarity with the target experimental XRPD pattern. The cell parameters were restrained (but not constrained) to those of the original CSP-generated crystal structure. The agreement between the form 4 powder pattern and that of the adjusted CSP-generated crystal structure was quantified in terms of a figure of merit (FOM), which is a value ranging from 0 to 1, with 1 being a perfect match. A plot of the relative TRHu(ST)23 lattice energies vs the FOM for the match with the experimental XRPD pattern of form 4 is shown in Supplementary Figure 4.

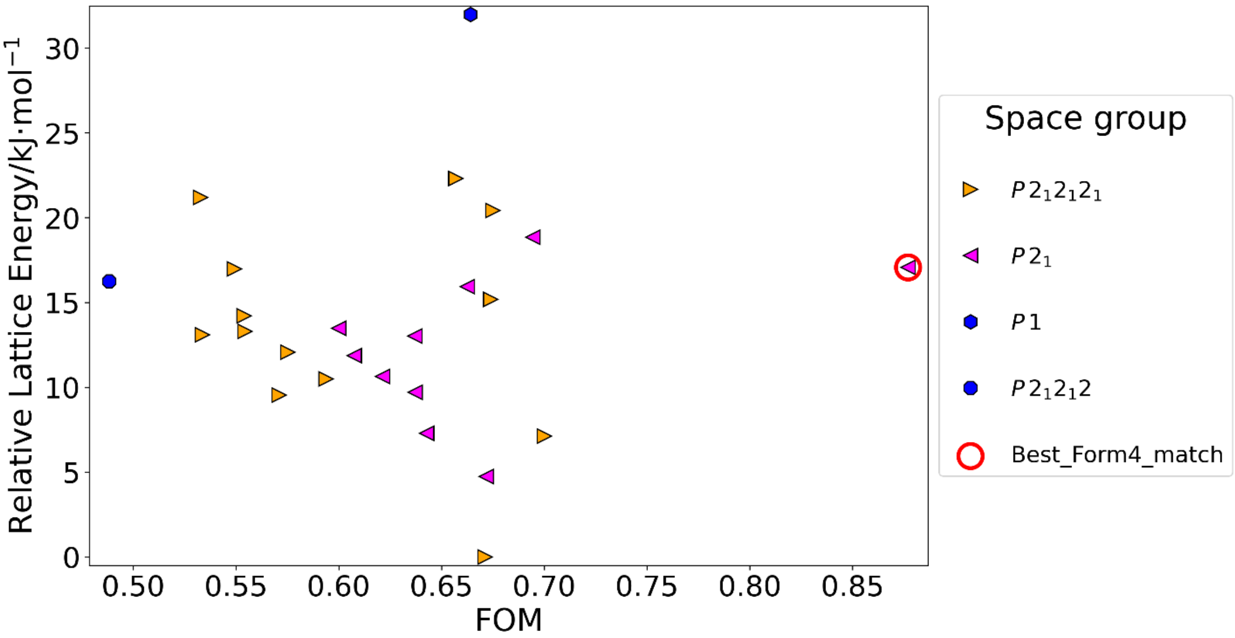

Supplementary Figure 4: Relative TRHu(ST)23 lattice energy of the DFT-D optimized CSP-generated crystal structures of ritonavir vs figure of merit for the match with the XRPD pattern of form 4. The best match in terms of FOM and energy is indicated by a red circle.

The crystal structure ranked 20th in terms of lattice energy, ~17.1 kJ·mol^{-1} above the global minimum, clearly stands out, having a significantly higher FOM than any of its competitors.

3.3 Three-dimensional electron diffraction

The crystalline sample, whose preparation is discussed above, was deposited on standard TEM grids (amorphous carbon on Cu) as a dry powder without grinding or other modification and measured on an ELDICO *ED-1* electron diffractometer at room temperature using the software ELDIX.[32] The device is equipped with a LaB_6 electron source operating at an acceleration voltage of 160 kV (λ = 0.02851 Å) and with a hybrid-pixel detector (Dectris QUADRO). The grid was screened for suitable crystals with needle-like shape in STEM (scanning transmission electron microscopy) mode and diffraction data was recorded in continuous rotation mode with a pseudo-parallel beam of ca. 750 nm diameter. A total of 135 crystals were measured, mostly having the same unit cell as known form 1, but 17 of them featured a different unit cell. Given the prior knowledge (see SI Section 3.1) that the crystalline sample contains a mixture of forms 1 and 4, these 17 crystals were assigned to form 4. From these, the 13 datasets with best quality (based on R_{int} values during data merging) were selected. Parts of the measurements affected by beam damage were omitted and the measurement details for all form 4 datasets used are given in Supplementary Table 1.

The data was processed using the APEX4 software package.[33] Frames were integrated separately for each crystal, then merged, scaled, and corrected for Lorentz effects, scan speed, background, and absorption using SAINT and SADABS.[34,35] Space group assignment was based on systematic absences, E statistics, and successful refinement of the structure. *Ab initio* structure solution with ShelXT or ShelXD failed, as the output in the correct space group did not show any recognizable molecule, but by using the predicted structure that best matched the XRPD of form 4 (lattice energy rank 20, see section above) as starting point a stable refinement could be achieved.[36,37] Structure solution was also possible using the simulated annealing method within SIR2019 by using the rigid molecular geometry from the predicted structure.[38] Least squares refinements within the kinematic approximation were carried out using ShelXL in conjunction with ShelXle by minimizing $\Sigma w(F_{obs}^2\text{-}F_{calc}^2)^2$ with the ShelXL weighting schemdise and using neutral electron scattering factors.[39-41] Non-hydrogen atoms were refined with anisotropic displacement parameters. Hydrogen atoms were placed in calculated positions based on typical distances for neutron diffraction and refined as a rigid rotating group with $U_{iso}(H) = 1.5 \cdot U_{eq}(C/O)$ for methyl and hydroxy groups and with a standard riding model and $U_{iso}(H) = 1.2 \cdot U_{eq}(C/N)$ for other groups. A split layer refinement was used for the disordered isopropyl group. Restraints on geometries and anisotropic displacement parameters were used to ensure convergence within physically reasonable limits. Deposition Number 2411739 contains the supplementary crystallographic data

for this paper, which is also include in Supplementary Data 2. These data are provided free of charge by the joint Cambridge Crystallographic Data Centre and Fachinformationszentrum Karlsruhe Access Structures service and can be accessed at www.ccdc.cam.ac.uk/structures.

Supplementary Table 1: 3D ED data collection details for ritonavir form 4 crystals used in refinement.

Crystal no.	Approximate size [µm]	Angular range [°]	Rotation per frame [°]	Exposure time [s]	Total exposure [s]	Frames measured	Frames used
1	2.1 x 1.0 x 0.4	−50 to +60	0.5	0.5	110	220	100
2	3.0 x 0.5 x 0.1	−50 to +30	0.5	0.5	80	160	60
3	2.6 x 0.6 x 0.2	−30 to +50	0.5	0.5	80	160	70
4	0.5 x 0.5 x 0.3	−45 to +45	0.5	0.5	90	180	50
5	4.9 x 1.0 x 0.3	−20 to +50	0.5	0.5	70	140	50
6	1.1 x 1.0 x 0.2	−40 to +40	0.5	0.5	80	160	55
7	3.0 x 1.3 x 0.3	−20 to +50	0.5	0.5	70	140	55
8	2.6 x 1.2 x 0.1	−30 to +50	0.5	0.5	80	160	40
9	2.2 x 0.8 x 0.3	−40 to +40	0.5	0.5	80	160	70
10	1.4 x 0.7 x 0.2	−30 to +30	0.5	0.5	60	120	35
11	2.0 x 0.8 x 0.2	−60 to 0	0.5	0.5	60	120	35
12	1.9 x 1.6 x 0.3	+20 to +70	0.5	0.5	50	100	20
13	5.0 x 1.5 x 0.3	0 to +60	0.5	0.5	60	120	30

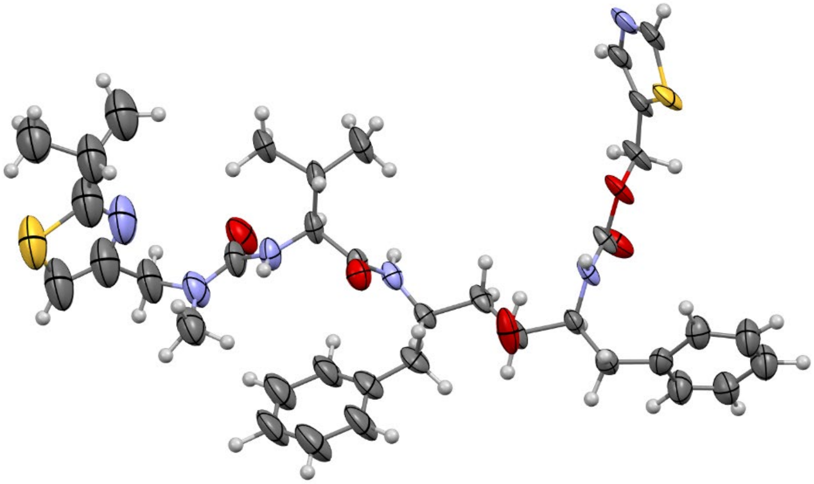

Supplementary Figure 5: Conformation of ritonavir in the crystal structure of form 4 with ellipsoids at the 50% probability level. Disorder of one isopropyl group is omitted. (Color code: C, dark grey; H, light grey; N, blue; O, red; S, yellow).

Supplementary Table 2: Structure and refinement details for ritonavir form 4.

**CCDC number**	2411739
**Empirical formula**	$C_{37}H_{48}N_6O_5S_2$
**Formula weight**	720.93
**Temperature [K]**	298
**Crystal system**	Monoclinic
**Space group (number)**	$P2_1$ (4)
***a* [Å]**	14.12(34)
***b* [Å]**	5.16(13)
***c* [Å]**	26.47(63)
***α* [°]**	90
***β* [°]**	96.67(5)
***γ* [°]**	90
**Volume [Å^3]**	1915
Z	2
**ρ_{calc} [g·cm^{-3}]**	1.250
**Crystal colour**	colourless
***θ* range [°]**	0.06−0.74
**Index ranges**	$-12 \le h \le 12$, $-4 \le k \le 4$, $-20 \le l \le 20$
**Reflections collected**	8726
**Independent reflections**	2142
**Completeness**	0.687
**Data / Restraints / Parameters**	2142 / 798 / 488
**Goodness of fit**	1.06
**Final R factors [I > 2σ(I)]**	$R_1 = 0.1457$, $wR_2 = 0.3406$
**Final R factors [all data]**	$R_1 = 0.1650$, $wR_2 = 0.3662$

As mentioned above, the 3D-ED solution of form 4 is disordered around the edge isopropyl group, with it taking two configurations, with occupancies of 58% and 42% respectively. This is like form 1 (YIGPIO02). One of the two disordered components was not generated by the CSP study (the other one is the structure matching lattice energy rank 20, discussed in the previous section), likely due to its significant instability, and was therefore optimized independently to place it on the crystal energy landscape.

Supplementary Note 4. CSP data analysis and further calculations

4.1 Calculation of the vibrational component of free energy.

Supplementary Figure 6 shows the static 0 K lattice energy landscape of ritonavir.

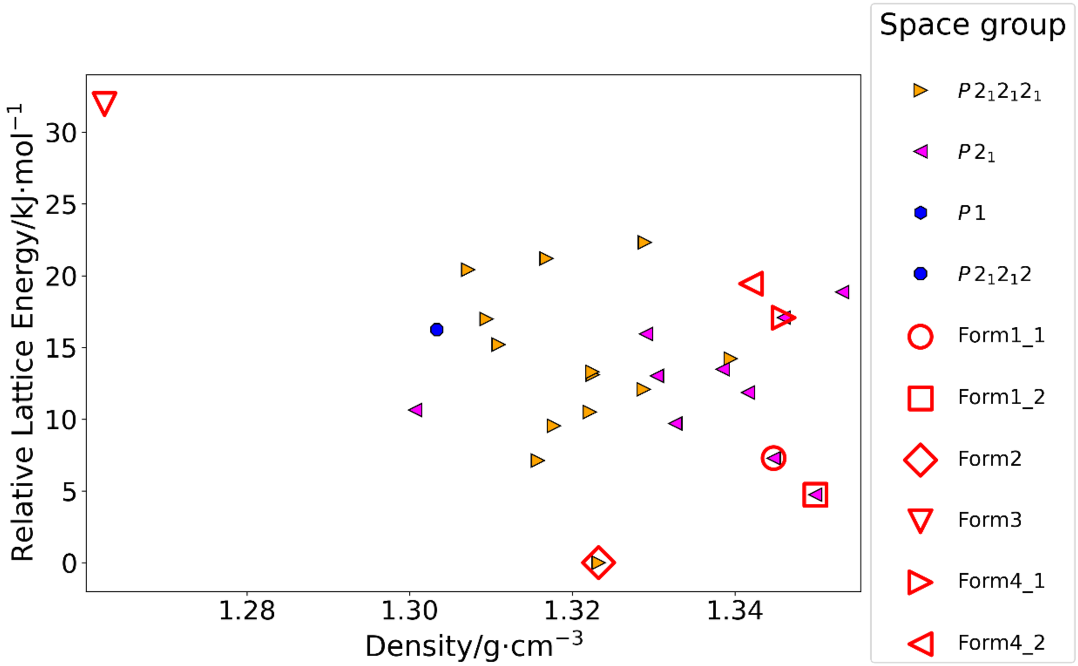

Supplementary Figure 6: Static 0 K lattice energy (relative to the global minimum) vs density plot summarizing the crystal energy landscape of ritonavir. Each point on the plot corresponds to a separate computer-generated crystal structure, labelled according to its space group. The structures matching form 2, as well as the disordered components of forms 1, and one of the disordered components of form 4, are indicated. Optimized form 3, as well as the optimized component of form 4 not found in CSP, are also shown.

However, the most relevant relative polymorphic stability in terms of pharmaceutical development is that calculated at room temperature, which can in some cases be different from the relative static lattice energies at 0 K.[42,43] Calculating the free energy at room temperature requires a calculation of the vibrational component to free energy ($F_{vib}(T)$) from the phonon frequencies; more detail can be found elsewhere.[16,43,44] Given the high computational cost associated with calculating the $F_{vib}(T)$ for all predicted polymorphs of ritonavir, the temperature dependent free energy was calculated at the PBE-NP level, according to the method outlined by Firaha *et al.*,[20] only for the ten predicted crystal structures of ritonavir lowest in TRHu(ST)23 lattice energies, as well as for optimized form 3 and for the two components of form 4. Note that only one of the form 4 disordered components had been generated in the CSP study, and the other

was optimized independently from the 3D-ED solution. Thus, for the remaining 13 ones the lattice energies were corrected by the average F_{vib} of the subset for which the vibrational calculations had been performed to estimate their free energies at finite temperatures and place them on the room temperature crystal energy landscape:[45]

$$A_{TRHu(ST)23\,E_{latt}\,only}(T) = E_{latt\,TRHu(ST)23} + \frac{\sum_{i=1}^{n} F_{vib}^{calculated}(T)}{n} \quad \text{(SE 1)}$$

Where n represents the number of structures for which $F_{vib}(T)$ has been explicitly calculated; in the case of ritonavir, *n*=13. This process is performed because other polymorphs of ritonavir will have $F_{vib}(T)$ values of a similar absolute size to those for which they had been explicitly computed, allowing a reasonable estimate of A(T) and thus to place them on a free energy landscape. Obviously, this estimate of $F_{vib}(T)$ is less accurate than an explicit calculation and will thus reduce the confidence level, which will be discussed later in this document.

4.2 Summary of the outcome of CSP study and computational expense.

In the crystal structure search step of the CSP, the maximum number of generated structures (10,000) was hit. When this happens, GRACE automatically reduces the size of the energy window for the first CSP step until it does not contain more than 10,000 crystal structures. Afterwards, 157 crystal structures were fully DFT-D optimized in the second intermediate re-ranking step and 24 were taken to the final step and further DFT-D optimized with tighter convergence settings. The TMFF accuracy was ~8.9 kJ·mol^{-1}.

The generation of the TMFF took ~78,400 CPU hours (up to 800 CPU cores at any time, ~4 days wall-clock time), the actual crystal structure prediction took ~1,461,600 CPU hours (up to 1,200 CPU cores at any time, ~51 days wall-clock time), the TRHu(ST)23 lattice energy calculations took ~207,600 CPU hours (up to 1,200 CPU cores at any time, ~5 days wall-clock time) and the $F_{vib}(T)$ calculations on the 13 predicted polymorphs took ~225,100 CPU hours (up to 1,200 CPU cores at any time, ~7 days wall-clock time).

4.3 Calculation of error bars.

The aforementioned recent publication by Firaha *et al.*[20] shows how to compute the error bars, representing one standard error, of relative finite temperature crystalline free energies as a function of number of non-water atoms (98 for ritonavir), the number of molecules in the asymmetric unit cell (Z')[46] and the number of water molecules (0, as only single-component crystal structures were considered). This formula was used to calculate the error bar for the 13 polymorphs for which the free energy calculations were performed. For the remaining 13, the

standard deviation of the explicitly calculated $F_{vib}(T)$ values was calculated and added to the intrinsic $\sigma\left(A^{explicit}_{TRHu(ST)23}(T)\right)$ by error propagation, as:

$$\sigma\left(A_{TRHu(ST)23\ E_{latt}\ only}(T)\right) = \sqrt{[\sigma\left(A^{explicit}_{TRHu(ST)23}(T)\right)]^2 + [\sigma\left(F^{calculated}_{vib}(T)\right)]^2} \qquad \text{(SE 2)}$$

The reasoning behind this is that if the explicitly calculated $F_{vib}(T)$ values are very similar to one another, then the vibrational component to free energy will have a smaller impact on the relative stabilities between polymorphs at finite temperature making the approximation of only computing the lattice energies less severe. The resulting free energy landscape at 300 K is shown in Supplementary Figure 7.

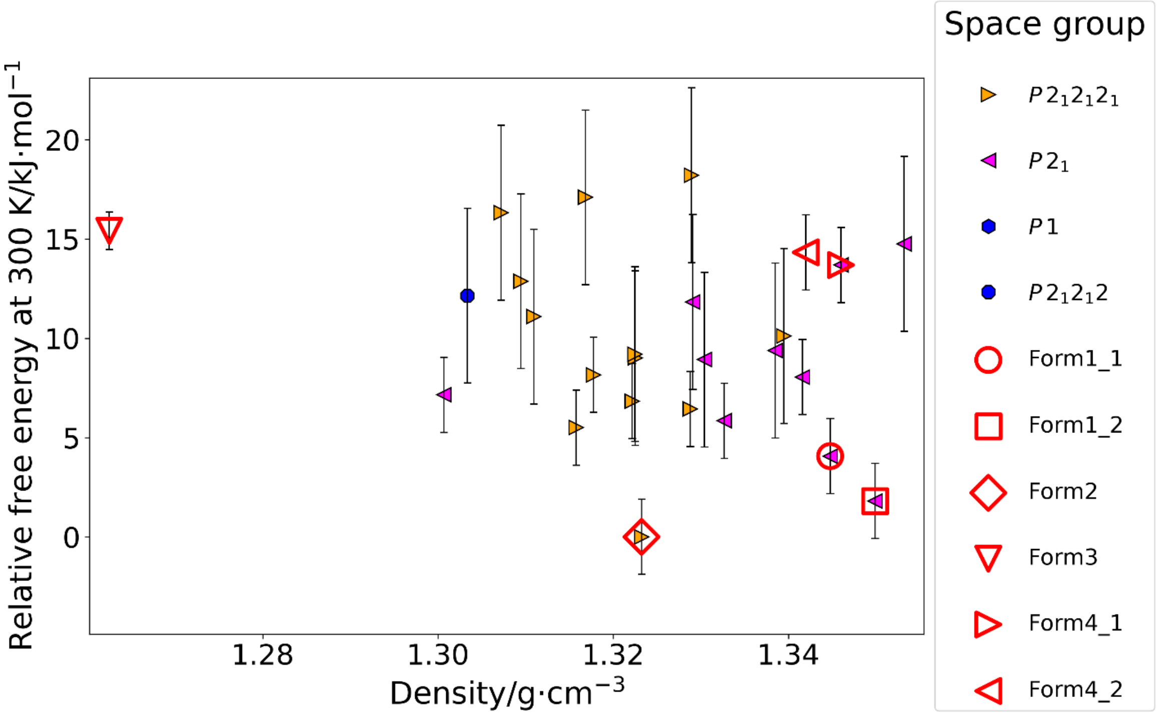

Supplementary Figure 7: Free energy at 300K (relative to the global minimum) vs density plot summarizing the crystal energy landscape of ritonavir. The free energies were calculated with the TRHu(ST)23 method. Each point on the plot corresponds to a separate computer-generated crystal structure, labelled according to its space group. The structures matching form 2, as well as the disordered components of forms 1, and one of the disordered components of form 4, are indicated. Optimized form 3, as well as the optimized component of form 4 not found in CSP, are also shown. More details about these crystal structures, as well on the 15-molecule root mean square distance ($RMSD_{15}$) with the experimental forms, can be found in SI Supplementary Table 3.

4.4 Calculation of disorder of the energetic effect of the forms 1 and 4 disorder.

As mentioned in the main manuscript and in the sections above, ritonavir forms 1 (YIGPIO02) and 4 are disordered around their respective edge isopropyl groups.[47] Therefore, there are two structures matching form 1 found in the CSP study: the more stable one is ranked 2nd in free energy at 300 K, 1.81 kJ·mol^{-1} above the global minimum rank 1 (matching form 2), and the less stable one being ranked 3rd, 4.07 kJ·mol^{-1} above the most stable predicted polymorph. On the other hand, only one structure matching form 4 was found in the CSP study, which is ranked 20th, 13.69 kJ·mol^{-1} above the global minimum rank 1; the other matching structure was not found likely due to its significant instability and, after it was optimized independently, it ended up being ranked 21st, 14.33 kJ·mol^{-1} above rank 1. Disorder can have a stabilizing energy effect by adding a configurational component to the free energy ($F_{config}(T)$).[48,49]

There are several ways to compute $F_{config}(T)$, which depend on the nature of the disorder, and on the strength of the interaction between the disordered groups.[48] The disordered iso-propyl groups are close to one another in both experimental forms 1 and 4 along the respective b-axes, but they are not likely to interact strongly and may be described in terms of isolated sites with configurations populated according to their Boltzmann distribution.[48] To verify this hypothesis, a 221 supercell (containing 8 molecules) of the more stable structure matching a disordered component of form 1, rank 2 (see Supplementary Figure 7), was generated, and one of the molecules was then replaced with the configuration contained in the less stable matching structure, rank 3. The same process was repeated for form 4, where a 231 supercell (containing 16 molecules) of the more stable structure matching a disordered component of form 4, rank 20, had one molecule replaced with the configuration contained in the less stable matching structure, rank 21. Then, these supercells were optimized at the PBE-NP level of theory with the same settings as in the final CSP stage. For form 1, the energy of the supercell is intermediate between those of the structures matching the disordered components of form 1 calculated at the same PBE-NP level of theory, indicating that the isolated-site disorder model is indeed appropriate to describe $F_{config}(T)$ for this system. For form 4, on the other hand, the supercell has a lower energy than either form by ~1 kJ·mol^{-1} and therefore a symmetry-adapted ensemble model would be needed to best describe the effect of disorder on its stability.[48] However, given the significant instability of the form 4 disordered components, we chose to apply an isolated-site model in this case too as the application of symmetry-adapted ensemble theory would carry a significant computational cost, would take a large amount to setup, and would not change the overall picture. Therefore, Equations 1 and 2 in the paper by Woollam *et al.*[48] were used to calculate the $F_{config}(T)$

for both system based on the free energy differences at 300 K computed with the accurate TRHu(ST)23 method (~2.2 kJ·mol^{-1} and ~0.6 kJ·mol^{-1} for forms 1 and 4 respectively), leading a F_{config} value of -0.19 kJ·mol^{-1} for form 1 and -0.58 kJ·mol^{-1} for form 4 at 300 K. Finally, $F_{config}(T)$ was added to the relative free energy of the more stable structure matching form 4 at 300 K to compute its overall stability as shown in Figure 2 in the main manuscript (see Supplementary Table 3 for more detail).

Note that the structure matching the minor configuration in YIGPIO02 is predicted to be more stable than that to the major one. Using Equation 4 in the manuscript by Woollam *et al*. the occupancy of major disordered component of YIGPIO02 is estimated to correspond to ~14%, *vs* an experimental one of ~56%.[47] However, the correct ratio, which would correspond to an energy difference in favor of the rank 3 crystal structure in Supplementary Figure 7 of ~0.2 kJ·mol^{-1} (vs the ~2.2 kJ·mol^{-1} against it coming from the TRHu(ST)23 calculations) is well within the confidence interval described by the error bars of the model. For form 4, the occupancy of the major disordered component is estimated to correspond to ~37% vs an experimental one of ~58%; once again the correct ratio, which would correspond to an energy difference in favor of the rank 21 crystal structure of ~0.4 kJ·mol^{-1} (vs the ~0.6 kJ·mol^{-1} against it coming from the TRHu(ST)23 calculations) is well within the expected error bar of the model.

Finally, a note about the error bars in Figure 2 in the main manuscript. The calculation of $F_{config}(T)$ is likely to further increase the confidence interval of the forms 1 and 4 disordered model relative to their competitors; this is especially true for form 4, where the isolated-site disorder model appears to be a less accurate approximation. However, as there is no validation for how disorder could affect the error bars, we decided to ignore it and just consider the ones associated with the TRHu(ST)23 calculations.

Supplementary Table 3: THRu(ST)23 free energies at 300 K (relative to the global minimum), structural and crystallographic parameters of the 24 unique crystal structures of ritonavir in the crystal energy landscape summarized in Supplementary Figure 7 and Figure 2 in the main manuscript. The disordered model of form 1 is highlighted in green, the structure matching form 2 in orange, optimized form 3 in blue, and the structure used as a starting model to refine form 4 in yellow. For the crystal structures in bold F_{vib} was explicitly calculated, for the rest it was estimated, as also reflected by the different sizes of the error bars.

Structure rank	ΔA at 300 K [kJ·mol^{-1}]	Error bar [kJ·mol^{-1}]	Density [g·cm^3]	Packing coefficient/ %	Space group	Z'	a/Å	b/Å	c/Å	α/°	β/°	γ/°	$RMSD_{15}$ with experimental /Å
**1**	**0.00**	**1.89**	**1.323**	**70.7**	**$P2_12_12_1$**	**1**	**9.81**	**18.60**	**20.43**	**90.00**	**90.00**	**90.00**	**0.154**
**2-3 Disorder**	**1.62 (1.81-4.07)**	**1.89, 1.89**	**1.350, 1.345**	**72.0, 71.54**	**$P2_1$, $P2_1$**	**1, 1**	**13.36, 13.39**	**5.23, 5.28**	**26.93, 26.76**	**90.00**	**103.63, 103.06**	**90.00**	**0.053, 0.038**
**4**	**5.51**	**1.89**	**1.316**	**70.3**	**$P2_12_12_1$**	**1**	**9.90**	**18.58**	**20.40**	**90.00**	**90.00**	**90.00**	/
**5**	**5.85**	**1.89**	**1.333**	**71.3**	**$P2_1$**	**1**	**13.42**	**5.21**	**27.06**	**90.00**	**102.28**	**90.00**	/
**6**	**6.44**	**1.89**	**1.329**	**70.9**	**$P2_12_12_1$**	**1**	**4.94**	**21.90**	**34.25**	**90.00**	**90.00**	**90.00**	/
**7**	**6.84**	**1.89**	**1.322**	**70.4**	**$P2_12_12_1$**	**1**	**9.67**	**14.58**	**26.47**	**90.00**	**90.00**	**90.00**	/
**8**	**7.15**	**1.89**	**1.301**	**68.8**	**$P2_1$**	**1**	**10.52**	**10.61**	**17.15**	**90.00**	**89.14**	**90.00**	/
**9**	**8.05**	**1.89**	**1.342**	**71.3**	**$P2_1$**	**1**	**13.46**	**5.21**	**26.95**	**90.00**	**102.84**	**90.00**	/
**10**	**8.16**	**1.89**	**1.318**	**70.3**	**$P2_12_12_1$**	**1**	**9.80**	**14.70**	**25.97**	**90.00**	**90.00**	**90.00**	/
11	8.93	4.40	1.330	70.9	$P2_1$	1	13.30	5.24	27.12	90.00	101.55	90.00	/
12	9.01	4.40	1.322	70.6	$P2_12_12_1$	1	9.82	14.60	25.99	90.00	90.00	90.00	/
13	9.21	4.40	1.322	70.6	$P2_12_12_1$	1	4.91	25.21	30.06	90.00	90.00	90.00	/
14	9.38	4.40	1.338	71.1	$P2_1$	1	13.34	5.26	27.14	90.00	76.13	90.00	/
15	10.11	4.40	1.339	71.4	$P2_12_12_1$	1	9.64	14.28	26.75	90.00	90.00	90.00	/
16	11.09	4.40	1.311	69.8	$P2_12_12_1$	1	9.83	18.74	20.53	90.00	90.00	90.00	/
17	11.83	4.40	1.329	70.8	$P2_1$	1	13.52	5.16	26.92	90.00	97.13	90.00	/
18	12.15	4.40	1.303	69.7	$P2_12_12$	1	19.20	38.53	5.10	90.00	90.00	90.00	/
**19**	12.88	4.40	1.309	69.8	$P2_12_12_1$	1	4.92	18.92	40.43	90.00	90.00	90.00	/
**20-21 Disorder**	**13.11 (13.69, 14.33)**	**1.89, 1.89**	**1.346, 1.342**	**71.7, 73.4**	**$P2_1$**	**1**	**13.71, 13.72**	**5.14, 5.10**	**26.43, 25.84**	**90.00**	**99.97, 99.55**	**90.00**	**0.489, 0.515**
22	14.76	4.40	1.353	71.9	$P2_1$	1	13.77	5.12	26.14	90.00	97.04	90.00	/
**23**	**15.42**	**0.95**	**1.262**	**68.9**	**P1**	**4**	**9.88**	**11.81**	**33.54**	**89.23**	**82.33**	**78.04**	**0.216**
24	16.33	4.40	1.307	69.6	$P2_12_12_1$	1	9.90	18.60	20.59	90.00	90.00	90.00	/
25	17.10	4.40	1.317	70.3	$P2_12_12_1$	1	9.78	13.85	27.66	90.00	90.00	90.00	/
26	18.22	4.40	1.329	70.8	$P2_12_12_1$	1	5.13	13.72	52.76	90.00	90.00	90.00	/

Supplementary References

1 Li, S. *et al.* Ritonavir Revisited: Melt Crystallization Can Easily Find the Late-Appearing Polymorph II and Unexpectedly Discover a New Polymorph III. *Molecular Pharmaceutics* **20**, 3854-3863, doi:10.1021/acs.molpharmaceut.2c00994 (2023).

2 Yao, X., Henry, R. F. & Zhang, G. G. Z. Ritonavir Form III: A New Polymorph After 24 Years. *Journal of Pharmaceutical Sciences* **112**, 237-242, doi:https://doi.org/10.1016/j.xphs.2022.09.026 (2023).

3 Spek, A. L. Single-crystal structure validation with the program PLATON. *Journal of Applied Crystallography* **36**, 7-13, doi:10.1107/S0021889802022112FILE: /proj/ads/abstracts/ (2003).

4 Spek, A. L. checkCIF validation ALERTS: what they mean and how to respond. *Acta Crystallogr E Crystallogr Commun* **76**, 1-11, doi:10.1107/s2056989019016244 (2020).

5 Sarma, B., Roy, S. & Nangia, A. Polymorphs of 1,1-bis(4-hydroxyphenyl)cyclohexane and multiple Z′ crystal structures by melt and sublimation crystallization. *Chemical Communications*, 4918-4920, doi:10.1039/B610323E (2006).

6 Neumann, M. A., Leusen, F. J. J. & Kendrick, J. A Major Advance in Crystal Structure Prediction. *Angewandte Chemie International Edition* **47**, 2427-2430, doi:10.1002/anie.200704247 (2008).

7 Neumann, M. A. Tailor-Made Force Fields for Crystal-Structure Prediction. *The Journal of Physical Chemistry B* **112**, 9810-9829, doi:10.1021/jp710575h (2008).

8 Grimme, S. Density functional theory with London dispersion corrections. *Wiley Interdisciplinary Reviews: Computational Molecular Science* **1**, 211-228, doi:10.1002/wcms.30 (2011).

9 Perdew, J. P., Burke, K. & Ernzerhof, M. Generalized Gradient Approximation Made Simple. *Physical Review Letters* **77**, 3865-3868, doi:10.1103/PhysRevLett.77.3865 (1996).

10 Perrin, M.-A., Neumann, M. A., Elmaleh, H. & Zaske, L. Crystal structure determination of the elusive paracetamol Form III. *Chemical Communications*, 3181-3183, doi:10.1039/B822882E (2009).

11 Kresse, G. & Joubert, D. From ultrasoft pseudopotentials to the projector augmented-wave method. *Physical Review B* **59**, 1758-1775, doi:10.1103/PhysRevB.59.1758 (1999).

12 Kresse, G. & Furthmüller, J. Efficiency of ab-initio total energy calculations for metals and semiconductors using a plane-wave basis set. *Computational Materials Science* **6**, 15-50, doi:https://doi.org/10.1016/0927-0256(96)00008-0 (1996).

13 Kresse, G. & Furthmüller, J. Efficient iterative schemes for ab initio total-energy calculations using a plane-wave basis set. *Physical Review B* **54**, 11169-11186, doi:10.1103/PhysRevB.54.11169 (1996).

14 TURBOMOLE V7.1 2016, a development of University of Karlsruhe and Forschungszentrum Karlsruhe GmbH, 1989-2007, TURBOMOLE GmbH, since 2007; available from http://www.turbomole.com.

15 Iuzzolino, L., McCabe, P., Price, Sarah L. & Brandenburg, J. G. Crystal structure prediction of flexible pharmaceutical-like molecules: density functional tight-binding as an intermediate optimisation method and for free energy estimation. *Faraday Discussions* **211**, 275-296, doi:10.1039/C8FD00010G (2018).

16 Mortazavi, M. *et al.* Computational polymorph screening reveals late-appearing and poorly-soluble form of rotigotine. *Communications Chemistry* **2**, 70, doi:10.1038/s42004-019-0171-y (2019).

17 Mattei, A. *et al.* Efficient Crystal Structure Prediction for Structurally Related Molecules with Accurate and Transferable Tailor-Made Force Fields. *Journal of Chemical Theory and Computation* **18**, 5725-5738, doi:10.1021/acs.jctc.2c00451 (2022).

18 Sohncke, L. & Teubner, B. G. *Entwicklung einer Theorie der Kristallstruktur*. viii, 247, 6 unnumberd pages : illustrations ; 23 cm (B.G. Teubner, 1879).
19 Grothe, E., Meekes, H. & de Gelder, R. Chirality and stereoisomerism of organic multicomponent crystals in the CSD. *CrystEngComm* **22**, 7380-7388, doi:10.1039/D0CE00403K (2020).
20 Firaha, D. *et al.* Predicting crystal form stability under real-world conditions. *Nature* **623**, 324-328, doi:10.1038/s41586-023-06587-3 (2023).
21 Blum, V. *et al.* Ab initio molecular simulations with numeric atom-centered orbitals. *Computer Physics Communications* **180**, 2175-2196, doi:https://doi.org/10.1016/j.cpc.2009.06.022 (2009).
22 Adamo, C., Cossi, M. & Barone, V. An accurate density functional method for the study of magnetic properties: the PBE0 model. *Journal of Molecular Structure: THEOCHEM* **493**, 145-157, doi:https://doi.org/10.1016/S0166-1280(99)00235-3 (1999).
23 Marom, N. *et al.* Many-Body Dispersion Interactions in Molecular Crystal Polymorphism. *Angewandte Chemie International Edition* **52**, 6629-6632, doi:https://doi.org/10.1002/anie.201301938 (2013).
24 Hermann, J. & Tkatchenko, A. Density Functional Model for van der Waals Interactions: Unifying Many-Body Atomic Approaches with Nonlocal Functionals. *Physical Review Letters* **124**, 146401, doi:10.1103/PhysRevLett.124.146401 (2020).
25 Řezáč, J., Greenwell, C. & Beran, G. J. O. Accurate Noncovalent Interactions via Dispersion-Corrected Second-Order Møller–Plesset Perturbation Theory. *Journal of Chemical Theory and Computation* **14**, 4711-4721, doi:10.1021/acs.jctc.8b00548 (2018).
26 Morissette, S. L., Almarsson, O. & Soukasene, S. Solvates and Polymorphs of Ritonavir and Methods of Making and Using the Same. Lexington, MA patent US7205413B2 (2007).
27 Hilden, J. L. *et al.* Capillary Precipitation of a Highly Polymorphic Organic Compound. *Crystal Growth & Design* **3**, 921-926, doi:10.1021/cg034061v (2003).
28 Morissette, S. L., Soukasene, S., Levinson, D., Cima, M. J. & Almarsson, Ö. Elucidation of crystal form diversity of the HIV protease inhibitor ritonavir by high-throughput crystallization. *Proceedings of the National Academy of Sciences* **100**, 2180-2184, doi:10.1073/pnas.0437744100 (2003).
29 Ankit, R. WebPlotDigitizer. **5.2** (2020).
30 Habermehl, S., Mörschel, P., Eisenbrandt, P., Hammer, S. M. & Schmidt, M. U. Structure determination from powder data without prior indexing, using a similarity measure based on cross-correlation functions. *Acta Crystallographica Section B* **70**, 347-359, doi:10.1107/S2052520613033994 (2014).
31 Zolotoyabko, E. Determination of the degree of preferred orientation within the March–Dollase approach. *Journal of Applied Crystallography - J APPL CRYST* **42**, 513-518, doi:10.1107/S0021889809013727 (2009).
32 ELDIX Software Suite, Version 5.5.3 (2024).
33 Apex Suite of Crystallographic Software, APEX4, Version 2022.1-1 (2022).
34 SAINT, Version 8.40B (2019).
35 SADABS, Version 2016/2 (2016).
36 Sheldrick, G. M. SHELXT - integrated space-group and crystal-structure determination. *Acta Crystallogr A Found Adv* **71**, 3-8, doi:10.1107/s2053273314026370 (2015).
37 Sheldrick, G. M. Experimental phasing with SHELXC/D/E: combining chain tracing with density modification. *Acta Crystallogr D Biol Crystallogr* **66**, 479-485, doi:10.1107/s0907444909038360 (2010).
38 Burla, M. *et al.* Crystal structure determination and refinement viaSIR2014. *Journal of Applied Crystallography* **48**, 306-309, doi:10.1107/S1600576715001132 (2015).

39 Sheldrick, G. Crystal structure refinement with SHELXL. *Acta Crystallographica Section C* **71**, 3-8, doi:10.1107/S2053229614024218 (2015).
40 Hubschle, C., Sheldrick, G. & Dittrich, B. ShelXle: a Qt graphical user interface for SHELXL. *Journal of applied crystallography* **44**, 1281-1284, doi:10.1107/S0021889811043202 (2011).
41 Peng, L. M. Electron atomic scattering factors and scattering potentials of crystals. *Micron* **30**, 625-648, doi:https://doi.org/10.1016/S0968-4328(99)00033-5 (1999).
42 Nyman, J. & Day, G. M. Static and lattice vibrational energy differences between polymorphs. *CrystEngComm* **17**, 5154-5165, doi:10.1039/C5CE00045A (2015).
43 Hoja, J. *et al.* Reliable and practical computational description of molecular crystal polymorphs. *Science Advances* **5**, eaau3338, doi:10.1126/sciadv.aau3338.
44 Hoja, J. & Tkatchenko, A. First-principles stability ranking of molecular crystal polymorphs with the DFT+MBD approach. *Faraday Discussions* **211**, 253-274, doi:10.1039/C8FD00066B (2018).
45 Zhu, X. *et al.* Interrogating the Solid–Solid Phase Transition Behavior of a Molecular Crystal with a Diverse Range of Solid-State Analytical Techniques and Computational Predictions. *Crystal Growth & Design* **24**, 8174-8180, doi:10.1021/acs.cgd.4c01078 (2024).
46 Steed, K. M. & Steed, J. W. Packing Problems: High Z' Crystal Structures and Their Relationship to Cocrystals, Inclusion Compounds, and Polymorphism. *Chemical Reviews* **115**, 2895-2933, doi:10.1021/cr500564z (2015).
47 Bauer, J. *et al.* Ritonavir: an extraordinary example of conformational polymorphism. *Pharm Res* **18**, 859-866, doi:10.1023/a:1011052932607 (2001).
48 Woollam, G. R., Neumann, M. A., Wagner, T. & Davey, R. J. The importance of configurational disorder in crystal structure prediction: the case of loratadine. *Faraday Discussions* **211**, 209-234, doi:10.1039/C8FD00072G (2018).
49 Habgood, M., Grau-Crespo, R. & Price, S. L. Substitutional and orientational disorder in organic crystals: a symmetry-adapted ensemble model. *Physical Chemistry Chemical Physics* **13**, 9590-9600, doi:10.1039/C1CP20249A (2011).
