# Supplementary material for: Predicting the ritonavir crisis by revisiting the polymorph landscape with crystal structure prediction and form 4 structure solution
Source: Commun Chem. 2025 Dec 22;8:404. doi: 10.1038/s42004-025-01814-6 (PMC12722319; doi:10.1038/s42004-025-01814-6)
Supplement: Supplementary file 3 — Description of Additional Supplementary Files [file 42004_2025_1814_MOESM3_ESM.pdf]

Description of Additional Supplementary Files:

File: Supplementary Data 1

Description: zip file contains a sub-folder (key_structures) containing the crystallographic information file (.cif) of the low energy crystal structure prediction generated structures of ritonavir optimized with the FHI-Aims code using DFT-D at the PBE-NP level of theory. The structures are named based on their ranks in Supplementary Table 3. For example the structure rank 1st in Supplementary Table 3 is called CSP_structure_rank_1.cif in the supplementary data folder, the one ranked 2nd is CSP_structure_rank_2.cif, and so on.

CSP_structure_rank_1.cif is a match to ritonavir form 2, CSP_structure_rank_2.cif and CSP_structure_rank_3.cif to the two disordered components of form 1, CSP_structure_rank_20.cif to one of the two disordered components of form 4. CSP_structure_rank_21.cif is the other disordered component of form 4 optimized from the experimental solution, while CSP_structure_rank_23.cif corresponds to optimized form 3.

File: Supplementary Data 2

Description: zip file contains the 3D-ED crystal structure solution of ritonavir form 4 in a .cif format.
